# Survival prediction in patients with gynecological cancer irradiated for brain metastases

**DOI:** 10.2340/1651-226X.2023.34899

**Published:** 2024-04-21

**Authors:** Silje Skjelsvik Os, Kjersti Skipar, Eva Skovlund, Ivar Hompland, Taran Paulsen Hellebust, Marianne Grønlie Guren, Kristina Lindemann, Esten Søndrol Nakken

**Affiliations:** a Department of Oncology, Oslo University Hospital, Oslo, Norway; b Department of Gynecological Oncology, Division of Cancer Medicine, Oslo University Hospital, Oslo, Norway; c Institute of Clinical Medicine, University of Oslo, Oslo, Norway; d Department of Oncology, Telemark Hospital Trust, Skien, Norway; e Department of Public Health and Nursing, Norwegian University of Science and Technology (NTNU), Trondheim, Norway; f Department of Medical Physics, Oslo University Hospital, Oslo, Norway

**Keywords:** Radiotherapy, brain metastases, ovarian cancer, endometrial cancer, cervical cancer, vulvar cancer, palliation

## Abstract

**Background and purpose:**

This large population-based, retrospective, single-center study aimed to identify prognostic factors in patients with brain metastases (BM) from gynecological cancers.

**Material and methods:**

One hundred and forty four patients with BM from gynecological cancer treated with radiotherapy (RT) were identified. Primary cancer diagnosis, age, performance status, number of BM, presence of extracranial disease, and type of BM treatment were assessed. Overall survival (OS) was calculated using the Kaplan–Meier method and the Cox proportional hazards regression model was used for multivariable analysis. A prognostic index (PI) was developed based on scores from independent predictors of OS.

**Results:**

Median OS for the entire study population was 6.2 months. Forty per cent of patients died within 3 months after start of RT. Primary cancer with the origin in cervix or vulva (*p* = 0.001), Eastern Cooperative Oncology Group (ECOG) 3–4 (*p* < 0.001), and the presence of extracranial disease (*p* = 0.001) were associated with significantly shorter OS. The developed PI based on these factors, categorized patients into three risk groups with a median OS of 13.5, 4.0, and 2.4 months for the good, intermediate, and poor prognosis group, respectively.

**Interpretation:**

Patients with BM from gynecological cancers carry a poor prognosis. We identified prognostic factors and developed a scoring tool to select patients with better or worse prognosis. Patients in the high-risk group have a particular poor prognosis, and omission of RT could be considered.

## Introduction

The incidence of brain metastases (BM) from solid cancers has increased over the recent decades [1]. Reasons for this development may include more frequent and better imaging techniques, prolonged survival due to improved systemic therapy regimens, and greater awareness among clinicians and patients [2]. Still, the occurrence of BM is a rare event, in particular in patients with gynecological cancers, with reported incidences rates of 0.3–0.9% in cervical cancer, 0.4–1.2% in endometrial cancer, 0.3–2.2% in ovarian cancer, and 0–0.7% in vulvar cancer [2–5].

The standard management of BM includes best supportive care (BSC) including steroids, surgical excision, and radiotherapy (RT). RT is administered as whole brain radiotherapy (WBRT), stereotactic radiotherapy (SRT), or partial brain radiotherapy (PBRT), solitary or in combination. Randomized controlled trials comparing WBRT with SRT for treatment of BM in solid cancers, have not found differences in overall survival (OS) [6, 7]. However, the well-known risk of developing cognitive deficit after WBRT [8], as well as the longer treatment time for delivering WBRT, increasing the patient burden, supports the use of SRT when feasible.

There are no current guidelines for treating BM in gynecological cancer patients, mainly due to the rare occurrence of BM, as well as the lack of large prospective trials. In 2022 and 2021, respectively, the American Society of Clinical Oncology-Society for Neuro-Oncology-American Society for Radiation Oncology (ASCO-SNO-ASTRO) [9] and The European Association of Neuro-Oncology – European Society for Medical Oncology (EANO-ESMO) [10] endorsed guidelines for treating BM from solid tumors, with specific sections for lung, melanoma, and breast. The general treatment recommendations found in these guidelines are often applied to gynecological cancer patients. EANO-ESMO recommends SRT [10] in patients with 1–4 BM, but it may also be considered in patients with a higher number of BM (5–10) if the cumulative volume is below 15 mL. In both guidelines, WBRT is favored in patients with multiple BM, and in patients where SRT is not feasible. Surgery is advised to patients with large tumors causing significant mass effect and in cases of single BM in patients with controlled systemic disease. Surgery is also considered in patients with cystic and necrotic BM due to an expected inferior response to SRT compared to more cell-dense tumors [10]. Both guidelines recommend postoperative SRT in all patients receiving surgery. For patients with a poor performance status, BSC alone is recommended.

Reflecting the low incidence of BM in gynecological cancers, published studies on the outcome after treatment for BM are exclusively retrospective analyses. A favorable prognosis has been reported for selected patients receiving multimodal treatment combining surgery, RT and chemotherapy [2, 11], as well as for patients presenting without extracranial disease at the time of BM diagnosis [2, 12]. However, firm conclusions from these results are difficult to draw due to retrospective studies with small sample sizes, and the lack of multivariable analysis correcting for relevant clinical variables [2, 11, 12]. Many gynecological patients with BM have a very short survival of less than 3 months [2, 11], questioning the benefit of treatment beyond BSC. In a retrospective study on gynecological cancer patients by Gressel et al. [11], a median OS of 9.0, 4.5, and 3.0 months for ovarian, endometrial, and cervical cancer respectively, was reported. Several prognostic scoring systems, across different cancers, have been developed to improve patient selection [13–16], and have identified performance status, number of BM, extracranial disease, and age as significant prognostic factors. However, none of these scoring systems are based on cohorts of patients with gynecological cancers.

Improved understanding of the expected survival benefit after treatment for BM in gynecological cancer patients may provide a more individualized treatment approach. Moreover, patients with an expected poor survival benefit will be spared from the treatment and treatment-related toxicity.

As a primary objective, we have reported survival outcomes in patients with BM from gynecological cancers treated with RT. As secondary objectives we have described treatment patterns of RT and we have developed a tool to support treatment decision-making in individual patients based on identified prognostic factors.

## Materials and methods

### Patient cohort

All patients with BM from gynecological cancer treated with RT from January 2006 to July 2021 at the Radium Hospital, Oslo University Hospital, were identified in the hospital’s RT registry. The hospital is a referral unit for the entire Health Region Southeast in Norway, with a population of 3.1 million citizens. Inclusion criteria included histologically verified primary diagnosis of ovarian-/tube-or peritoneal cancer, endometrial cancer, cervical cancer, or vulvar cancer, and treatment with RT for BM. Exclusion criteria included presence or history of another primary cancer and cancer of unknown origin.

Details of the RT regimens were extracted from the same registry. The diagnosis of BM was verified by computed tomography (CT) or magnetic resonance imaging (MRI). Survival data were available through linkage to statistics Norway.

Scanning of the brain is not routinely performed in gynecological cancer patients. CT and/or MRI is carried out in case of suspicious symptoms and therefore we consider that all patients in this study have likely been symptomatic at the time of diagnosis. After diagnosis of BM, patients are usually given corticosteroids until after completed RT.

To explore any potential change in treatment patterns during the study period, we divided the period into equally sized halves: 2006–2013 and 2014–2021.

### Ethics

This study was considered a quality assurance project by the Regional Committee for Medical Research Ethics. A waiver for ethical approval was granted, and a signed informed consent from patients was not required. Recommendations of processing personal data or health information were obtained from the hospital’s data-protection officer.

### Statistical analysis

Clinical variables were identified and categorized as follows: Age at time of BM diagnosis (≤65 vs >65 years), primary cancer diagnosis (ovarian vs endometrial vs cervical and vulvar), ECOG performance score (0–1 vs 2 vs 3–4), number of brain lesions (single vs 2–4 vs >4), extracranial disease at time of BM diagnosis (no vs yes), and type of BM treatment (WBRT alone vs SRT alone vs surgery combined with RT). OS was calculated from the first day of RT of BM, and until death of any cause or end of follow-up, November 29th 2022. Other medical data were extracted from the hospital’s electronic medical records. Differences between groups were compared using the log rank test, and presented as Kaplan–Meier curves. Hazard ratios (HRs) with 95% confidence intervals were calculated using the Cox proportional hazards regression model.

Significant clinical variables were included in the multivariable age-adjusted analysis. Variables with an independent association with OS, including primary tumor site, ECOG status, and extracranial disease, were used to create a prognostic index (PI) based on the Cox model estimated coefficients and corresponding confidence interval. The diagnoses of cervical cancer and vulvar cancer were grouped into one category because of the low number of vulvar cancers and assumed similar biology. Based on sum scores from the PI described earlier, three risk groups were generated. All data were analyzed using SPSS version 28.0 (SPSS Inc., USA). *P*-values less than 0.05 were regarded statistically significant.

## Results

### Patient characteristics and treatment for BM

In total 146 patients were eligible for the study. Two patients were lost to follow up, leaving 144 patients eligible for the analyses.

The clinical and treatment characteristics of the study population are presented in Table 1. The primary cancer diagnosis was ovarian cancer in 69 (48%) of the patients, endometrial cancer in 43 (30%), cervical cancer in 29 (20%), and vulvar cancer in 3 (2%) patients. The median age at diagnosis of BM was 65 years (range 24–91) for the whole group, and 67 years (range, 24–86) for ovarian, 69 years (range 46–91) for endometrial, 49 years (range, 27–72) for cervical, and 64 years (range, 60–71) for vulvar cancer. The median time from initial diagnosis to BM was 35.7 months (range, 0–250) for ovarian cancer, 21.3 months (range 0–276) for endometrial cancer, 25.5 months (range 0.8–212) for cervical cancer, and 132 months (range 18–145) for vulvar cancer. Only one patient presented with BM at the time of primary diagnosis. Single BM was present in 33% of the patients. Extracranial disease was present in 83% of the patients at the time of diagnosis of BM.

**Table 1:** Patient and treatment characteristics.

Factor	Ovarial (*n* = 69)		Corpus (*n* = 43)		Cervix (*n* = 29)		Vulva (*n* = 3)		Total (*n* = 144)	
	*n*	%	*n*	%	*n*	%	*n*	%	*n*	%
Age										
≤65	30	43	14	33	27	93	2	67	73	51
>65	39	57	29	67	2	7	1	33	71	49
ECOG										
0–1	33	48	19	44	14	48	1	33	67	47
2	11	16	8	19	10	34	1	33	30	21
3–4	25	36	16	37	5	17	1	33	47	33
Number of lesions										
1	24	35	16	37	8	28	–	–	48	33
2–4	13	19	17	40	6	20	3	100	39	27
>4	32	46	10	23	15	52	–	–	57	40
Extracranial disease										
No	18	26	4	9	3	10	–	–	25	17
Yes	51	74	39	91	26	90	3	100	119	83
Treatment										
WBRT alone	44	64	28	65	17	59	3	100	92	64
SBRT alone	14	20	9	21	7	24	0	0	30	21
Surgery+RT	11	16	6	14	5	17	0	0	22	15

For the 72 patients treated between 2006 and 2013, 17% received SRT. For the patients treated between 2014 and 2021, this percentage increased to 40%. SRT was delivered with a wide range of doses and fractionation schedules depending on tumor size, number of BM, and location. For 11 patients, WBRT was delivered in five fractions of 4 Gray (Gy), while 76 patients received 10 fractions of 3 Gy. In 11 patients (7.5%), the planned RT schedule was interrupted before completion due to deteriorating performance status.

For the 22 (15%) patients who underwent surgery for BM, the addition of RT was administered as either SRT, partial brain RT, or WBRT. Due to recurrence of BM, 17 patients received two series of RT and one patient received three series of RT.

### Outcome

**Figure 1:**
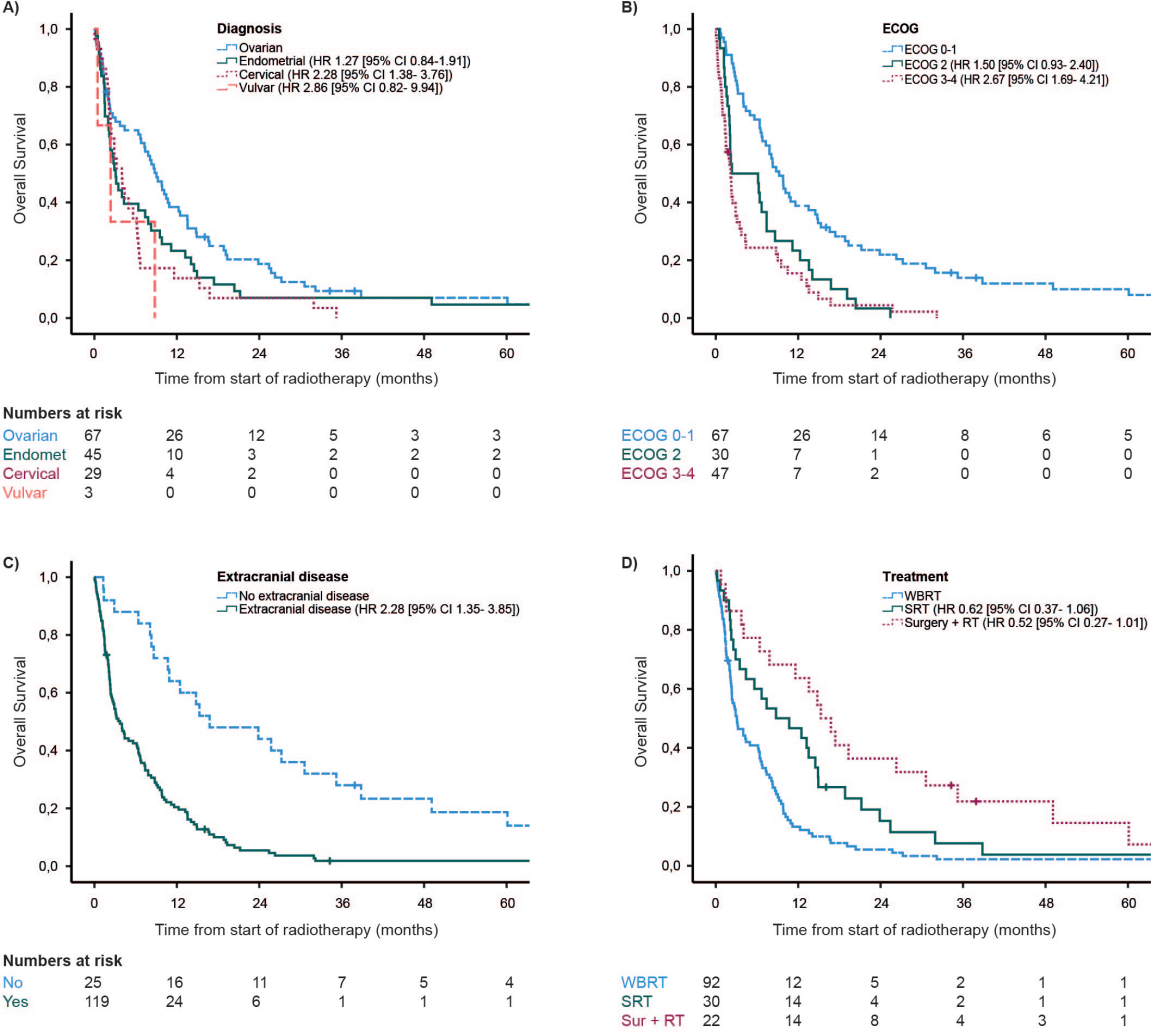
Overall survival. (A) Patients with primary diagnosis of ovarian, endometrial, cervical, and vulvar cancer. (B) Patients with ECOG status of 0–1, 2, and 3–4. (C) Patients with or without extracranial disease at the time of brain metastases diagnosis. (D) Patients treated with WBRT, SRT, or surgery followed by WBRT or SRT. WBRT: whole brain radiotherapy; SRT: stereotactic radiotherapy.

At the time of last follow up, only six patients were still alive. The median OS for the total study population was 6.2 months (range 0–172 months), with 3 months- and 1 year OS rates of 60 and 28%, respectively. The diagnosis-specific median OS was 9.0, 3.1, 4.0, and 2.4 months for ovarian, endometrial, cervical, and vulvar cancer, respectively (Figure 1a). Median OS was 9.2, 6.3, and 2.2 months for patients with ECOG 0–1, 2, and 3–4, respectively (Figure 1b). Patients without extracranial disease had a median OS of 16.8 months compared to 3.5 months in patients with extracranial disease (Figure 1c). Median OS of patients treated with WBRT was 3.1 months compared to 8.8 months in the SRT group and 15.3 months in the surgery + RT group; however, these findings were not statistically significant (Figure 1d).

In multivariable analysis, primary cervical – or vulvar cancer (HR 2.28; CI 95% 1.38–3.76; *p* = 0.001), ECOG 3–4 (HR 2.67; CI 95% 1.69–4.21; *p* < 0.001), and the presence of extracranial disease (HR 2.28; CI 95% 1.35–3.85; *p* = 0.001) were significantly associated with shorter OS. SBRT (HR 0.62; CI 95% 0.37–1.06; *p* < 0.08) and surgery before WBRT or SBRT (HR 0.52; CI 95% 0.27–1.01; *p* = 0.05) compared to WBRT was close to, but not statistically significant (Table 2).

**Table 2:** Uni- and multivariable Cox proportional hazards model for overall survival.

Factor	Univariable analysis		Multivariable analysis		Prognostic index (PI) model	
	HR (95% CI)	P	HR (95% CI)	P	Coefficient (95% CI)	Score
Age						
≤65 (*n* = 73)	1.00		1.00			
>65 (*n* = 71)	1.22 (0.87–1.71)	0.24	0.98 (0.66–1.47)	0.93		
Primary tumor						
Ovarian (*n* = 69)	1.00		1.00		0	0
Endometrial (*n* = 43)	1.48 (1.00–2.19)	0.05	1.27 (0.84–1.91)	0.25	0.24 (–0.16–1.44)	0.5
Cervical (*n* = 29)	1.77 (1.13–2.77)	0.01	2.28 (1.38–3.76)	0.001	0.77 (0.31–1.23)	1
Vulva (*n* = 3)	2.62 (0.82–8.42)	0.11	2.86 (0.82–9.94)	0.10		
ECOG						
0–1 (*n* = 67)	1.00		1.00		0	0
2 (*n* = 30)	2.11 (1.34–3.32)	0.001	1.50 (0.93–2.40)	0.10	0.53 (0.07–0.98)	0.5
3–4 (*n* = 47)	2.84 (1.90–4.24)	<0.001	2.67 (1.69–4.21)	<0.001	1.02 (0.60–1.44)	1
Extracranial disease						
No (*n* = 25)	1.00		1.00		0	0
Yes (*n* = 119)	3.30 (2.02–5.41)	<0.001	2.28 (1.35–3.85)	0.002	0.97 (0.46–1.48)	1
No. brain lesions						
1 (*n* = 48)	1.00		1.00			
2–4 (*n* = 39)	1.41 (0.91–2.18)	0.13	0.77 (0.46–1.27)	0.30		
>4 (*n* = 57)	1.63 (1.09–2.43)	0.02	0.97 (0.57–1.65)	0.92		
Treatment						
WBRT alone (*n* = 92)	1.00		1.00			
SBRT alone (*n* = 30)	0.54 (0.35–0–83)	0.005	0.62 (0.37–1.06)	0.08		
Surgery + WBRT/SBRT (*n* = 22)	0.34 (0.20–0.57)	<001	0. 52 (0.27–1.01)	0.05		

Univariable and multivariable analysis of factors associated with overall survival in patients with brain metastases, and the prognostic index (PI) model, including regression coefficient with 95% confidence intervals (CI) and corresponding chosen scores

**Figure 2:**
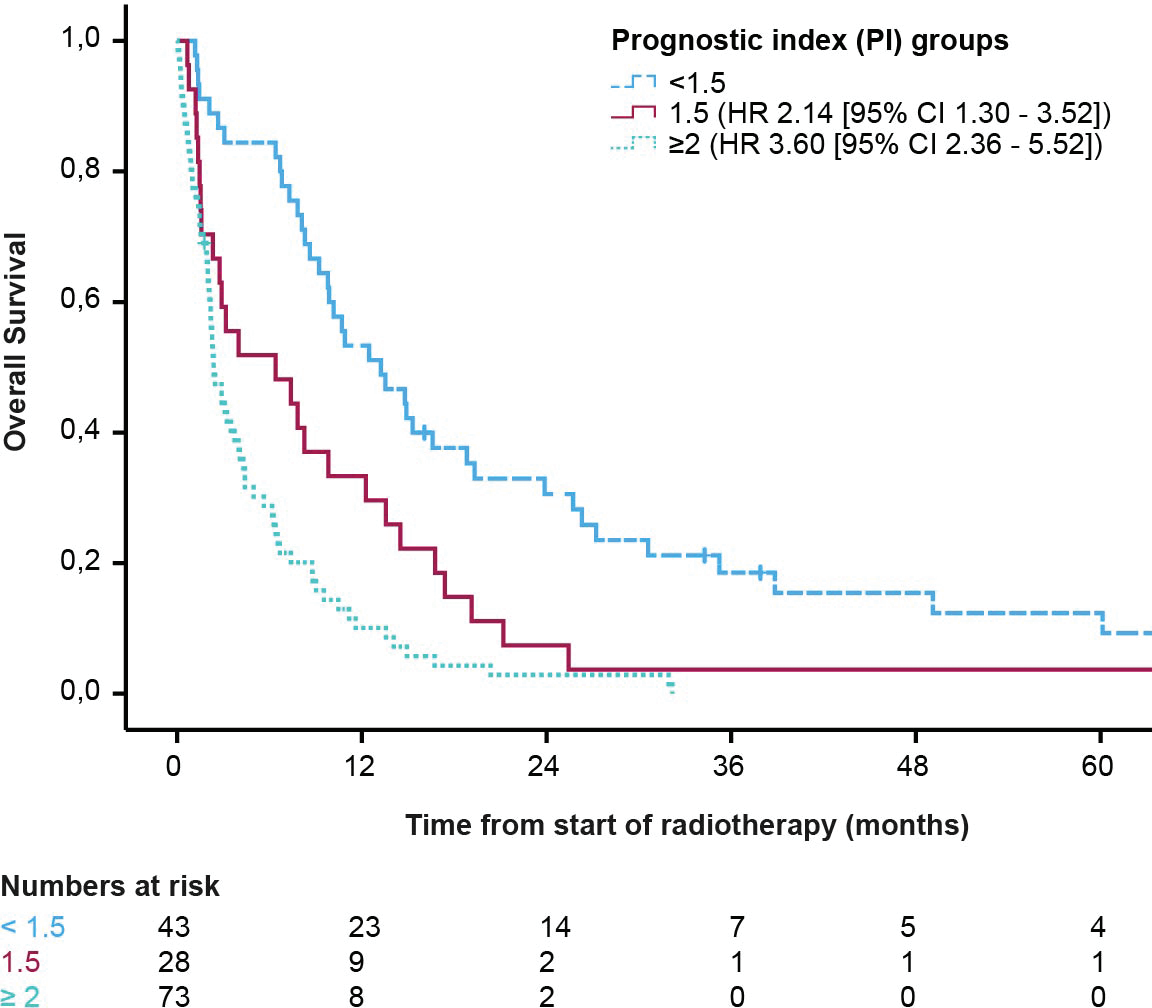
Overall survival according to the three defined prognostic groups. <1.5 points (*n* = 45), 1.5 points (*n* = 28), and ≥2 points (*n* = 71).

A PI was developed to distinguish between patients according to prognosis. Significant prognostic variables in multivariable analysis were assigned with 0, 0.5, or 1 point in the following way: Primary diagnosis of endometrial cancer = 0.5 point, primary diagnosis of cervical or vulvar cancer = 1 point, ECOG 2 = 0.5 point, ECOG 3–4 = 1 point, and presence of extracranial disease = 1 point. Primary diagnosis of ovarian cancer, ECOG 0–1 and no extracranial disease were assigned 0 points. The sum scores could range between 0 and 3 points, and were then grouped into three categories: <1.5, 1.5, and ≥ 2.0. The scores were associated with distinct differences in risk of death with a HR 2.14; CI 95% 1.30–3.52; *p* = 0.003 for patients with a risk score of 1.5 and a HR of 3.61; CI 95% 2.36–5.52; *p* < 0.001 for a score of ≥2. The median OS for the three different prognostic groups were 13.5 (CI 95% 8.13–18.93), 4.0 (CI 95% 0–9.83), and 2.4 (CI 95% 1.65–3.09) months, with the poorest survival in patients with a score ≥2 (Figure 2). A risk table is presented in Table 3.

**Table 3:** Prognostic crosstable.

Primary diagnosis	ECOG 0–1		ECOG 2		ECOG 3–4	
	No extracr. disease	Extracranial disease	No extracr. disease	Extracranial disease	No extracr. disease	Extracranial disease
Ovarian	0	1	0.5	1.5	1	2
Endometrial	0.5	1.5	1	2	1.5	2.5
Cervical + Vulvar	1	2	1.5	2.5	2	3

Crosstable of prognostic factors to aid decision making in whether or not to radiate patients with brain metastases from gynecological cancer. Green (<1.5 point) = best prognosis, radiation recommended, yellow (1.5 points) = medium prognosis, radiation can be considered, orange (≥2.0) = bad prognosis, radiation is not recommended.

Scores are assigned in the following way: Primary diagnosis of endometrial cancer = 0.5 point, primary diagnosis of cervical or vulvar cancer = 1 point, ECOG 2 = 0.5 point, ECOG 3–4 = 1 point, and presence of extracranial disease = 1 point. Primary diagnosis of ovarian cancer, absence of extracranial disease and performance status of ECOG 0–1 gives 0 points.

## Discussion

This retrospective single-center study confirmed the poor prognosis of patients with BM from primary gynecological cancer with a median OS for the whole cohort of 6.2 months. Forty per cent of patients died within 3 months after start of RT and only 28% of the patients survived beyond 12 months. This emphasizes the high number of patients receiving RT within the last 3 months of their life and where BSC only should be preferred [9, 10].

Our findings are in accordance with what has been reported in patients with other solid cancers. Preliminary results from a large prospective study of 930 patients with BM from different solid cancers showed that the vast majority of patients treated with WBRT lived shorter than 3 months [17].

Randomized clinical trials investigating BM in other cancers have not found differences in OS when comparing WBRT and SRT [6–8]. Nasu et al. [2] concluded that aggressive multimodal treatment is warranted in the treatment of BM from gynecological cancers in carefully selected patients. We support the recommendation regarding the possible benefit from a multimodal treatment approach in selected patients, even though our data regarding this point only reached close to statistical significance.

As reported by others [18], we see an increasing use of SRT in treating BM over time. Patients treated with SRT increased from 17 to 40% from the first to the second half of the study period. We anticipate that the number of patients treated with SRT will increase even further in the years to come. Although no statistically significant difference in OS between the different treatment groups was found in our study, less treatment-related cognitive impairment [8–10] and a shorter overall treatment time, favors SRT in feasible patients.

We found that cancer origin, ECOG performance status, and the presence of extracranial disease were significantly associated with OS. This is in accordance with preliminary findings from another large Norwegian prospective study where ECOG 0–1, age <65, and non-progressive extracranial disease prior to BM diagnosis are reported as factors associated with longer OS in WBRT-treated patients [17]. Previous retrospective studies on gynecological cancer patients have identified different factors associated with poor survival, including active extracranial disease, multiple BMs, old age, poor performance status, single modality treatment, and cervical/endometrial cancer [2, 11, 12, 19–21]. These studies differ in size and for some, only ovarian cancer patients are included. This might explain the inconsistency in findings regarding the above-mentioned factors. There is for example conflicting evidence regarding multiple BM as an independent negative prognostic factor [3, 22, 23]. In our study, multiple BM was not found to be independently associated with prognosis.

In this study, we present a PI for patients with BM from gynecological cancers treated with RT. The PI may support decision-making in individual patients. Based on this index, we have created a categorized risk table to further enhance clinical applicability. However, the PI is based on the limited sample size of patients who had undergone RT, and needs further validation. Rades et al. [24] suggested a similar prognostic system, including ECOG and presence or absence of extracranial disease, and recommended different fractionation regimens according to the prognostic group. Our risk table only provides assessment of expected survival benefit, but is based on a significantly larger, population-based cohort, which increases general validity. Recommendations of different fractionation regimens would be valuable for the clinician. However, the change in treatment over time, with increased use of SRT, makes this challenging, and prospective studies are warranted.

Regarding the choice of treatment modality, we refer to the existing general guidelines by ASCO-SNO-ASTRO and EANO-ESMO [9, 10]. The risk table may, however support the decision on whether to initiate or omit from active treatment. We lack data on patients treated with BSC alone, as well as patients treated only with surgery, which would have added value to the study. It is reasonable to assume that parallels can be drawn from studies investigating BM in other cancers. Nieder et al. compared WBRT (total dose of 20 or 30 Gy) to BSC in 113 patients with BM from different primary tumors and adverse prognostic factors. Median OS for all patients was 2 months, with no significant difference between BSC and 20 Gy. A significant improvement from median 1.7 months to median 2.2 months was observed in the 30 Gy WBRT group [25]. This is in line with the findings in the QUARTZ trial. In this prospective randomized study, BSC versus WBRT was compared in patients with metastases from non-small cell lung cancer that was inoperable and unsuitable for SRT. There was no evidence of difference in OS, overall quality of life, or use of dexamethasone between the two groups [26]. Based on our risk table, patients with a high sum score, and hence the poorest prognosis, would most likely benefit from omission of active treatment, and could be advised to BSC alone.

A major strength of the study is the inclusion of a population-based cohort that contains a significant number of unselected patients. Furthermore, we are providing a prognostic risk table based on simple parameters that easily could be applied in prospective trials. Ideally, prognostic models should be developed through three main phases: model development, external validation, and investigation of impact in clinical practice. As in our study, the majority of publications simply discuss model building, a small number disclose external validation, and very few take clinical implication into account [27]. Since the incidence of patients with BM from gynecological patients is low, external validation would require collaborative, multicenter studies to ensure adequate patient recruitment. There are limitations of our study. Due to low incidence of patients with vulvar cancer, these patients were assigned the same category as cervical cancers. For patients with vulvar cancer, the PI has limited value. Furthermore, RT was delivered with heterogenous fractionation schedules. In addition, we lack data on quality of life and symptom burden, similar to studies on other cancer diagnoses [25, 26]. Future studies should not only assess survival benefit, but also patient-reported outcomes measures and symptom burden to provide information valuable to treatment decision-making.

Eastern Cooperative Oncology group (ECOG) performance status was inconsistently reported in the medical records. When not reported, the first author made an estimate based on notes from nurses and doctors at the time of start of RT of BM. Misclassification is thus possible; however, most patients were admitted to the hospital, and the estimate of ECOG status was therefore based on thorough observations.

In conclusion, our study highlights the short life expectancy in patients with gynecological cancer and BM, and describes prognostic factors for survival. These findings may support clinicians in their treatment decision. The PI is easily applicable in prospective clinical trials for further validation.

## Data Availability

The data that support the findings of this study are available on request from the corresponding author, SSO. The data are not publicly available due to privacy of research participants.
